# New biochip immunofluorescence test for the serological diagnosis of pemphigus vulgaris and foliaceus: A review of the literature^☆^^☆☆^

**DOI:** 10.1016/j.ijwd.2017.10.001

**Published:** 2018-02-03

**Authors:** Rachel R. Xuan, Anes Yang, Dedee F. Murrell

**Affiliations:** Department of Dermatology, St George Hospital, Sydney, New South Wales, Australia; University of New South Wales, Sydney, New South Wales, Australia

## Abstract

The immunoassays that are available for the serological diagnosis of the more common subtypes of autoimmune blistering diseases such as pemphigus vulgaris (PV) and pemphigus foliaceus (PF) include enzyme-linked immunosorbent assay (ELISA) testing to specific antigens desmoglein (Dsg)1 and Dsg3, direct immunofluorescence (DIF), indirect immunofluorescence (IIF), and immunoblotting. A review of the literature on the biochip assay was conducted. Six studies investigated the validity of a new biochip, mosaic-based, IIF test in patients with pemphigus and demonstrated its relatively high sensitivity and specificity (Dsg3: 97.62-100%, 99.6-100%; Dsg1: 90%, 100%) in comparison with ELISA (Dsg3: 81-100%, 94-100%; Dsg1: 69-100%, 61.1-100%), and/or IIF (PV: 75-100%, 91.8-100%; PF: 67-100%) using suitable substrates. So far, validation studies of the biochip have been conducted in four countries (Germany, Italy, Turkey, and Poland) but none in the southern hemisphere. Caucasian patients were recruited as normal controls for these studies; thus, the diagnostic value of the biochip remains uncertain in population groups of other ethnicities. A range of disease control patients were recruited including patients with linear immunoglobulin A dermatosis, psoriasis, discoid lupus erythematosus, lichen planus, and noninflammatory skin diseases (e.g., squamous cell carcinoma, basal cell carcinoma, and vascular leg ulcers). Prospective studies with control patients from a diverse range of ethnicities are needed to better validate the biochip.

## Introduction

Pemphigus describes a group of potentially life-threatening, autoimmune, bullous diseases that affect the skin and mucous membranes. Autoantibodies are directed against cutaneous desmosomal glycoproteins (cadherins) that connect neighboring keratinocytes, which results in the loss of cell-to-cell adhesion (acantholysis; Mahoney et al., 1999). This manifests clinically as intraepithelial blisters.

Autoantibodies target Dsg3 (130 kDa) in patients with mucosal pemphigus vulgaris (PV), Dsg1 (165 kDa) in patients with pemphigus foliaceus (PF), and both in patients with a mucocutaneous form of PV (Amagai et al., 1999, Amagai et al., 1999, Kershenovich et al., 2014). There are two major types of classical pemphigus, including PV where acantholysis occurs in the suprabasal spinous layer and PF where acantholysis occurs in the subcorneal granular layer (Goncalves et al., 2011).

The worldwide incidence of pemphigus is one to five patients per million persons per year. However, occurrence varies greatly between geographical regions with higher incidences of PV in the Indian subcontinent, the Mediterranean region, and the Middle East and particularly in Arabic and Iranian populations (Ruocco et al., 2013).

Individuals with certain human leukocyte antigen (HLA) allotypes are predisposed to PV. However, the degree of susceptibility differs depending on ethnic origin. HLA-DRB1*0402 is associated with the disease in Ashkenazi Jews but DRB1*1401/04 and DQB1*0503 are associated with non-Jewish European and Japanese patients, respectively (Bystryn and Rudolph, 2005). The sporadic form of PF is most common in Europe and the United States and associated with HLA DRB1*0102 and 0404, but the endemic form (i.e., fogo selvage) occurs in certain regions of Brazil and Morocco with the same susceptibility gene (HLA DRB1*0102; Moraes et al., 1991). Pemphigus affects both men and women with a slight preponderance in female patients and manifests clinically in patients ages 20 to 40 years (Zhao and Murrell, 2015).

**Fig. 1:**
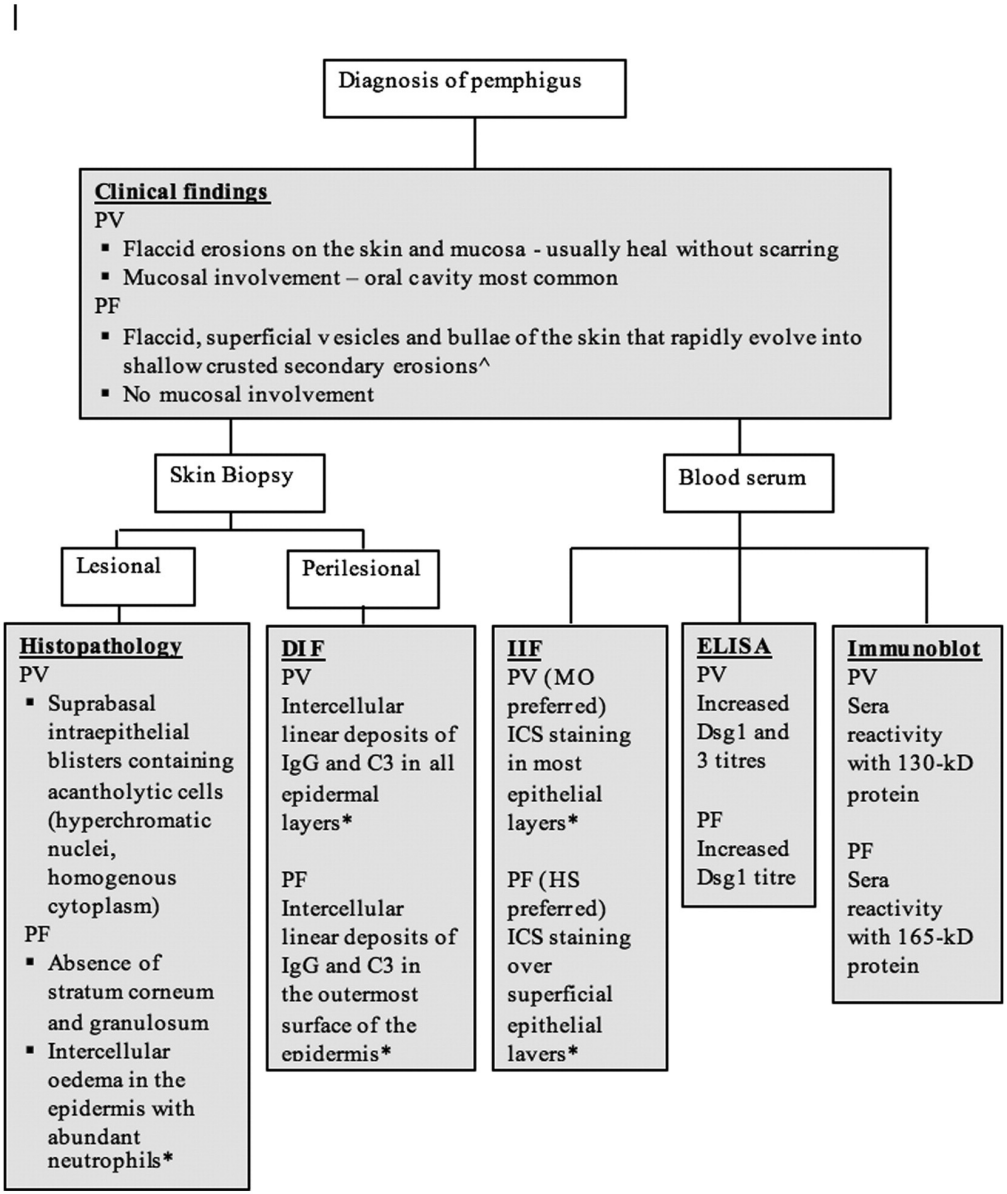
Conventional multistep approach to diagnosing pemphigus vulgaris and pemphigus foliaceus.*(Mascaro, 2015). ^(James et al., 2011).

In Australia, the process of diagnosing pemphigus involves typical physical findings, histopathology, direct immunofluorescence (DIF), indirect immunofluorescence (IIF), and enzyme-linked immunosorbent assay (ELISA) testing to Dsg1 and Dsg3. Although a multistep approach (Fig. 1) is used to diagnose pemphigus, the current diagnostic gold standard is the visualization of autoantibodies in the skin or mucous membranes by DIF of perilesional skin biopsy tissue (Schmidt and Zillikens, 2010). IIF is a well-established screening tool to circulate autoantibodies. The results of IIF tests for pemphigus antibodies is significantly influenced by the type of substrate that is used. PV sera reacts strongly with monkey esophagus, and PF sera prefers guinea pig esophagus (Sabolinski et al., 1987).

**Fig. 2:**
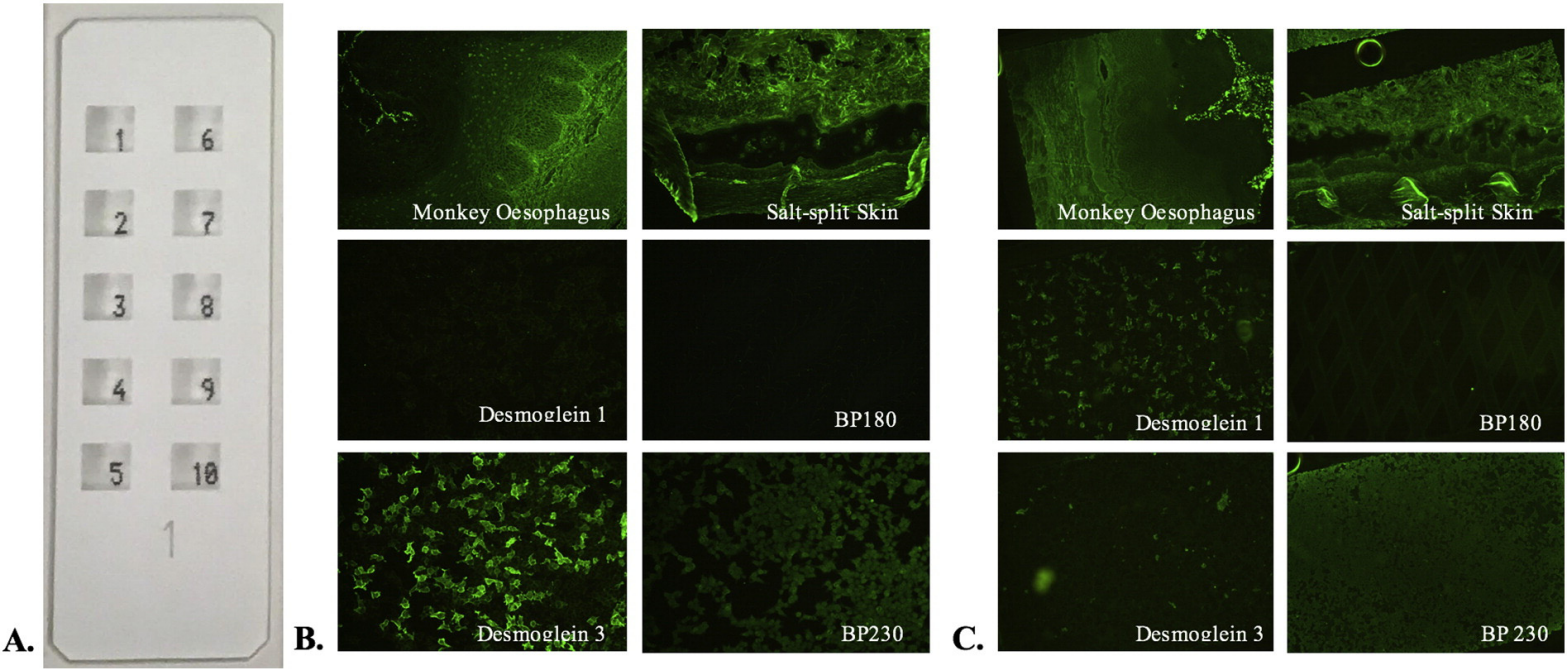
(A) Biochip slide with 10 incubation fields, each with six different biochips. (B) Immunofluorescence staining that is positive for pemphigus vulgaris. (C) Immunofluorescence staining that is positive for pemphigus foliaceus. [Format adapted from van Beek et al., 2012.]

In the 1990s, ELISA testing was developed with recombinant human Dsg1 and Dsg3 proteins to detect circulating autoantibodies (Weiss et al., 2015). ELISA values to Dsg3 are thought to correlate with disease activity in patients with pemphigus (Mortazavi et al., 2009, Sharma et al., 2006) and serve as a predictive means to assess disease severity (Daneshpazhooh et al., 2007).

Although a relatively easy method, immunoblotting (IB) has a low sensitivity due to the denaturing of protein antigens during electrophoresis (Tsuruta and Hashimoto, 2015). Here, we evaluate the evidence for a new biochip, mosaic-based, IIF technique that combines the screening of and targets recombinant antigenic substrates in a single miniature incubation field.

## Diagnostic tools for pemphigus

### Clinical and histological diagnosis

PV is virtually always associated with mucosal lesions, which appear most commonly in the oral cavity. However, patients can also develop erosions or blisters on the skin, which are known as the mucocutaneous form of PV (Schmidt et al., 2015). PF is not associated with mucosal erosions; however, patients may still manifest dry eye syndrome (Tan et al., 2015). Patients with PF develop flaccid, fragile superficial vesicles, and bullae of the skin that deroof quickly and appear more commonly as shallow secondary erosions (James et al., 2011).

A histopathological diagnosis for pemphigus is based on the results from a lesional skin biopsy test. The tissue sample is placed in 10% formalin for storage and transport and later paraffin-embedded and stained in hematoxylin and eosin stain (Schmidt et al., 2015, Suliman et al., 2013). In patients with PV, suprabasal intraepidermal blisters contain acantholytic cells that have hyperchromatic nuclei and homogenous cytoplasm. The cells of the basal layer remain attached to the basement membrane zone (BMZ) by their hemidesmosomal attachments and produce a characteristic appearance that looks like a row of tombstones. On the other hand, PF is usually suspected when there is an absence of stratum corneum and granulosum and the presence of neutrophilic spongiosis (i.e., intercellular edema in the epidermis with abundant neutrophils; Mascaro, 2015).

### Direct immunofluorescence

DIF microscopy requires a perilesional biopsy of normal skin (i.e., at least 1 cm away from the inflamed area) or mucosa that is placed in isotonic saline solution or Michel’s medium or frozen liquid nitrogen to avoid denaturation of the protein antigens (Aoki et al., 2010). It is important not to take a biopsy across the edge of a blister and cut it in half for DIF because the inflamed edge may have consumed the antibodies to be detected, which leads to a false negative result.

The specimen is sectioned in a cryostat into 4 to 6 μm frozen sections. These are incubated with fluorescence-tagged antibodies that are directed against human immunoglobulin (Ig) G, IgM, IgA, C3 fraction of complement, and fibrinogen (Mascaro, 2015). After several washes, the unbound antibodies are removed and the section is examined under a fluorescence microscope. In both types of pemphigus, there are intercellular deposits of IgG and C3 on the edges of keratinocytes, which gives a fishnet-like pattern. In patients with PV, these deposits are found in all epidermal layers, but in patients with PF, they exist predominantly in the outermost surface of the epidermis (Aoki et al., 2010).

### Indirect immunofluorescence

IIF allows for the detection of circulating autoantibodies against epithelial antigens by incubating patient serum with appropriate commercially available substrates, such as monkey esophagus or human skin, that contain the target antigen (Kanitakis, 2001). A tissue section of the epithelial substrate is incubated with patient serum at initial dilutions of 1:10 or 1:20 for 30 minutes and then washed to remove the unbound antibodies (Kanitakis, 2001, Mascaro, 2015). Titers can be determined by doubling these dilutions to 1:40, 1:80, and 1:160. Sections are then incubated with antibodies that are directed against IgG (or IgA when IgA pemphigus is suspected) conjugated with fluorescein isothiocyanate dye and examined with a fluorescence microscope (Mascaro, 2015).

The discrepancy in sensitivities between the epithelial substrates that are preferred by PV and PF is presumably because of the higher expression of Dsg3 and Dsg1 among monkey esophagus and human skin, respectively (Harman et al., 2000, Harman et al., 2000). PV sera produces a characteristic fishnet-like pattern on most epithelial layers but PF sera pattern appears predominantly over the superficial epithelial layers; however, IIF appearances in patients with PV and PF are often indistinguishable (Kanitakis, 2001).

### Enzyme-linked immunosorbent assay

In recent years, recombinant proteins of the full length of extracellular domains of Dsg3 and Dsg1 have been produced by the baculovirus expression system, which allows for the detection of circulating autoantibodies and serological differentiation between mucosal dominant PV, mucocutaneous PV, and PF (Ishii et al., 1997, Tsuruta and Hashimoto, 2015). The incubation of patient sera, which is added to microwells on Perspex plates that are coated with recombinant Dsg3 and Dsg1 proteins, allows the antibodies to react with the antigens. After washing to remove unbound antibodies, horseradish peroxidase conjugated IgG and peroxidase substrate is added (Chan, 2005). The enzyme activity of these peroxidases is detected via incubation with chromogen (Tsuruta and Hashimoto, 2015). The optical density of each well is measured automatically by a microtiter plate reader at 495 nm.

Quantitative ELISA titers have been shown to correlate with disease activity as well as the clinical transition between PF and PV (Komai et al., 2001). The difference between the two commercially available ELISAs is that EuroImmun (Lubeck, Germany) employs the recombinant protein using conformational ectodomains of Dsg1 and Dsg3 that are produced in the human embryonic kidney (HEK) 293 cells to maintain the greatest similarity between the posttransitional protein with human epidermal proteins, but the mannose binding lectin (MBL) assay uses conformational ectodomains that are generated by baculovirus expression in Hi5 insect cells (Tampoia et al., 2012).

### Immunoblotting

In IB, human epidermal extracts are denatured using chemicals and heat to unfold the proteins that are then separated according to molecular weight with gel electrophoresis. The proteins are transferred onto a nitrocellulose or polyvinylidene difluoride membrane that is overlaid with patient sera and then with peroxidase-conjugated antihuman IgG and IgA. The reactions between patient IgG antibodies with the 130 kDa Dsg3 in patients with PV and 160 kDa Dsg1 in patients with PF are visualized as separate bands on blotting paper against standard ladder proteins of certain molecular weights (Tsuruta and Hashimoto, 2015).

### Biochip

The biochip (Dermatology Mosaic 7, EuroImmun, Lubeck, Germany) method combines the screening of autoantibodies and target antigen-specific substrates in a single miniature incubation field. On a standard-sized biochip slide, there are 10 incubation fields and each has a mosaic of six different substrates (Fig. 2): frozen section of monkey esophagus; frozen section of human 1M NaCl-split skin; HEK293 cells that are transfected with pTriEx-1 constructs of Dsg1 protein ectodomain (amino acids 1-569); HEK293 cells that are transfected with pTriEx-1 constructs of Dsg3 protein ectodomain (amino acids 1-640); HEK293 cells that are transfected with a C-terminal globular domain of constructs of bullous pemphigoid (BP) 230 protein (amino acids 1875-2649); and microdrops of NC16A-4X BP180-free antigen (amino acids 490-562) that are expressed in *Escherichia coli* (Tampoia et al., 2012, van Beek et al., 2012).

Slides are incubated with serial serum dilutions of 1:10 in phosphate-buffered saline (PBS Tween) for 30 minutes and then rinsed with and immersed in PBS Tween for 5 minutes. Fluorescein isothiocyanate-conjugated goat antihuman IgA/G is applied for 30 minutes at room temperature to detect bound antibodies and then washed and examined with a fluorescence microscopy (Russo et al., 2014, van Beek et al., 2012).

On the monkey esophagus sections, intercellular staining occurs when pemphigus antibodies are directed against desmosomes and react with antigens on the surface of keratinocytes, which displays a granular fluorescence of the intercellular matter in the whole stratum spinosum. On salt-split skin sections, BMZ staining indicates BMZ autoantibodies that are directed against the epidermal basal membrane and produces a linear fluorescent pattern between the stratum basale and connective tissue, which is indicative of pemphigoid disease or epidermolysis bullosa acquisita (EBA).

A positive result of PF, PV, and BP is identified by cytosolic granular fluorescence on the other biochips that are coated with recombinant Dsg1, Dsg3, and BP230 antigens, respectively. However, this positive pattern is present only in some cells (i.e., transfected cells) because these cells are mixed with nontransfected cells to discriminate among true-positive samples with antibodies against recombinant antigens and those with antibodies against other cytosolic antigens. In the biochips that contain NC16A-4X BP180 free antigen, positive reactivity is characterized by diamond-shaped fluorescent microdrops. A negative result is obtained when samples display a homogenous reactivity in both the transfected and nontransfected cells (Tampoia et al., 2012).

## Objective

This report systematically evaluates the current literature with regard to the diagnostic value of the biochip in comparison with the IIF and ELISA methods in patients with PV and PF.

## Methods

### Literature sourcing

A systematic search for journal articles that compared the sensitivity and specificity of diagnostic tests for patients with PV and PF was conducted in four online databases, three of which were via the Ovid SP search interface: MEDLINE (1946 to 2017), Cochrane (2005 to 2017), and EMBASE (1974 to February 2017). Language was not limited to English and the following search terms were used separately and in combination: “pemphigus”, “biochip”, “ELISA”, “immunofluorescence”, “sensitivity”, “specificity”, “Desmoglein 1”, and “Desmoglein 3”. The reference lists of selected articles were also examined for further relevant articles.

### Inclusion and exclusion criteria

The studies that were included had to be a comparison of diagnosis tools that were used in the confirmation of PV and PF in patients (i.e., IIF, IB, DIF, and ELISA tests). Articles that were animal trials, specific case reports, poster presentations, or were based solely on pemphigoid or correlating diagnostic tools with disease activity were excluded.

### Review

Articles were initially screened by titles and then abstracts in accordance with the inclusion and exclusion criteria. Duplicate articles were removed, and the remaining articles were critically appraised.

## Results

The literature search generated 2234 articles, of which 74 were deemed relevant. Among the relevant articles, seven studies investigated the validity of the new biochip immunofluorescence test and have been critically appraised based on their strengths and limitations.

Van Beek et al. (2012) from Germany attempted to validate the biochip IIF technique and demonstrated a high sensitivity and specificity for IIF microscopy on monkey esophagus (100% for PV; 98% for PF; overall specificity 89.1% for pemphigus), anti-Dsg3 reactivity (98.5%, 100% for PV), and anti-Dsg1 reactivity (90%, 99.6% for PF). Although the study recruited a relatively large sample size of 42 patients with BP, 65 patients with PV, 50 patients with PF, 97 disease and 100 healthy control patients, the research was limited by a low case-to-control ratio (< 1:2) as well as hospital-based recruitment of control patients with other dermatological conditions such as linear IgA bullous dermatosis, vascular leg ulcers, basal cell carcinoma, and squamous cell carcinoma, which leads to a potential source of selection bias. However, the statistical significance of the results was improved with the systematic use of clinical findings, positive DIF, and Dsg3 or Dsg1 ELISA tests to confirm patient diagnoses.

Similarly, a diagnostic algorithm was applied in the second component of the prospective study that compared the biochip mosaic with the conventional multistep procedure as described by Schmidt and Zillikens (2011). The results revealed a high diagnostic agreement in 425 of 454 patient sera (93.6%; k-value 0.88; van Beek et al., 2012) and high concordance for the diagnosis of PV and PF as reflected by k values of 0.91 and 0.88, respectively. All sera were anonymized before being analyzed by experienced staff members of EuroImmun and the Dermatology Department at the University of Lubeck in Germany, which reduced potential reader bias. The same company was responsible for the development of the biochip; thus, the staff of the diagnostic laboratories at EuroImmun analyzed the sera, which might have introduced financial bias.

Furthermore, patient sera were obtained consecutively from a single site of recruitment and is therefore not representative of the entire population. The study also demonstrated that immunofluorescence titers of the novel biochip paralleled both ELISA values and disease activity. However, this was carried out on a small sample size (three patients with PV and three patients with PF) at nonstandardized timepoints throughout the disease course. Furthermore, scoring was performed retrospectively on the basis of photographs and patient records; thus, there may have been lesions that were simply not documented in the clinical records or images.

Later during the same year, an Italian study conducted by Tampoia et al. (2012) investigated the diagnostic accuracy of the new IIF multiplex biochip method by comparing two commercially available ELISA tests (MBL, Nagoya, Japan and EuroImmun, Lubeck, Germany). The biochip showed a high diagnostic sensitivity and specificity for patients with PV (100%, 100%), but the integrated circuits pattern on monkey esophagus substrate demonstrated a lower sensitivity and specificity (83.3%, 95.5%) compared with that reported by van Beek et al. (2012).

An excellent interrater agreement among the methods for anti-Dsg3 autoantibodies was observed (IIF multiplex vs. EuroImmun ELISA; k value 0.967; IIF multiplex vs. MBL ELISA, k value 1.000). A good observer agreement between the two ELISA methods were also observed for anti-Dsg3 autoantibodies (k value 0.967; Tampoia et al., 2012). Although patient sera were collected in three clinical laboratories, the study was conducted on a small sample size of 36 patients with PV and 40 patients with BP, who were all from one site of recruitment, which further reduced the statistical significance of the results. Furthermore, only 54 disease (n = 38 psoriasis, n = 16 discoid lupus erythematosus or lichen planus) and 40 healthy control patients were recruited, which resulted in a relatively poor ratio of approximately 1.24 controls per case.

Another Italian study by Russo et al. (2014) investigated the diagnostic use of the biochip for the serological diagnosis of PV and revealed a sensitivity of 97.62% and specificity of 100%. A major downfall of this study as well as past biochip validation studies was the lack of diversity when patients were recruited. The majority of the patients was of Caucasian background, which rendered the results highly specific and invalid for other population groups. There were insufficient controls to match the number of PV cases; however, the diagnosis was previously established via clinical features as well as histological and immunopathological findings (notably DIF and serum detection of autoantibodies by MBL ELISA). Although the sample size was considerably smaller including 42 patients with PV as well as 10 healthy and 10 disease control patients (n = 9 BP; n = 1 EBA), its results were comparable with previous studies.

The novel biochip has also been used to investigate the prevalence of pemphigus autoantibodies in the general population. Prussmann et al. (2015) from Germany reported that 0.31% (95% confidence interval [CI], 0.18-0.44%) of 7063 healthy blood donors had autoantibodies against pemphigus, 0.21% (95% CI, 0.11-0.32%) with anti-Dsg1 reactivity, and 0.10% (95% CI, 0.03-0.17%) with anti-Dsg3 reactivity. The EuroImmun ELISA validation of these findings confirmed 7 of 15 Dsg1 IIF positive samples and 6 of 7 Dsg3 IIF positive samples (Prussmann et al., 2015). A large sample size was recruited and careful measures were taken to avoid duplicate testing with further collection restricted to first-time donors; however, the study had the same limitations as previous studies including the use of a single site for recruitment. This is particularly detrimental to this study as the selection of donors was meant to act as a representation of the general healthy population.

A more recent study from Turkey that was conducted by Uzun et al. (2016) also investigated the diagnostic value of this new biochip method and recruited 45 patients with pemphigus, 18 patients with BP, and 35 control patients with skin diseases that were not specified. In patients with pemphigus, the sensitivity and specificity of the mosaic-based immunofluorescence test was considerably high (91%, 97%) and a good rate of agreement was observed between the biochip and ELISA tests (p < 0.01; Uzun et al., 2016). A downfall of the study was that no healthy control patients were recruited. Furthermore, the data did not differentiate between patients with PV and those with PF.

Russo et al. (2017) conducted another Italian study to evaluate the use of a biochip to detect anti-Dsg autoantibodies in salivary samples for a diagnosis of PV. A high concordance rate between the biochip and the ELISA test for serum was observed, but there was a lack of correlation between the serum and the salivary samples by both diagnostic tests (Russo et al., 2017). As with their previous study, there was a lack of diversity in the recruitment of patients (n = 8, Caucasian patients with PV). The results of this study were also statistically insignificant and inconclusive due to the small sample size and almost nonexistent number of control patients (n = 2, normal healthy individuals; n = 1, EBA; n = 1 BP). On the other hand, salivary anti-Dsg1 and Dsg3 ELISAs have been tested to reveal high specificities of 98.9% (Mortazavi et al., 2015), and salivary Dsg1 antibodies showed a significant correlation with mucosal severity (Hallaji et al., 2010).

The latest study was conducted in Poland and was the first to compare the original IIFc biochip method with a modified version (IIFm) to diagnose patients with pemphigus (Gornowicz-Porowska et al., 2017). The modified test replaced the usual fluorescein-conjugated anti-human IgG with a mouse monoclonal antihuman fluorescein-conjugated IgG4 secondary antibody to assess IgG4 antibodies to pemphigus antigens. Their results demonstrated a higher sensitivity and specificity for IIFm (Dsg1: 100%, 100%; Dsg3: 100%, 78%, respectively) compared with IIFc that support the use of IIFm to improve the diagnostic accuracy of pemphigus. A positive association was also found between the IIFc and ELISA methods (p < 0.05); however, no comparison was made between IIFm and ELISA. The downfalls of this study were the limited number of patients with PV and the absence of control patients to validate the biochip. Furthermore, the study concludes that IgG4, although diagnostically significant, is inconsistently detected with the modified IIF.

## Discussion

Zhou et al. (2016) reported that the sensitivity and specificity of the histological finding of acantholysis (66%, 100%) is higher than that of clinical features (50%, 96%); however, both are inferior to that of DIF (89%, 99%; Helander and Rogers, 1994). This is further supported by Mysorekar et al. (2015), who demonstrated that DIF had a sensitivity of 98.1% for the diagnosis of pemphigus. Furthermore, a good concordance rate between clinical, histological, and DIF diagnoses has been reported in immune-mediated skin disorders (observed agreement = 93.4%; k = 0.90 with 95% CI, 0.86-0.94.

The relatively recent development of IIF and ELISA tests have further facilitated the diagnosis of autoimmune bullous dermatoses and comparable sensitivities and specificities have been reported (PV: 75-100%, 91.8-100%; PF: 67-100% for IIF; Dsg3: 81-100%, 94-100%; Dsg1: 69-100%, 61.1-100% for ELISA; Amagai et al., 1999, Amagai et al., 1999, Barnadas et al., 2015, Bracke et al., 2013, Chiang et al., 2015, Cozzani et al., 1994, Daneshpazhooh et al., 2007, Daneshpazhooh et al., 2014, Hallaji et al., 2010, Harman et al., 2000, Harman et al., 2000, Ishii et al., 1997, Jiao and Bystryn, 1997, Khandpur et al., 2010, Mortazavi et al., 2015, Schmidt et al., 2010, Sharma et al., 2006, Tampoia et al., 2012, van Beek et al., 2012, van Beek et al., 2017, Zagorodniuk et al., 2005). An international, multicenter study further demonstrated a high diagnostic agreement of 93.6% (Cohen k value, 0.95) between DIF results and multivariant ELISA testing for patients with pemphigus (van Beek et al., 2017). IB is a very specific but highly skilled, time-consuming technique that can only be carried out in appropriately equipped research laboratories. In comparison with other diagnostic tools, IB is less sensitive, particularly in patients with PF (PF, 43%; PV, 83%), which is possibly due to the destruction of pemphigus antibodies that are directed against conformational epitopes during the electrophoresis, thus, rendering the antigen nonreactive with its antibody (Jiao and Bystryn, 1997).

In addition to its high sensitivity and specificity, the biochip is also known for the simplicity of its execution as the reagents come in a convenient kit that does not require sophisticated equipment or expert laboratory technicians. However, in Australia, the conventional procedure used to diagnose pemphigus is through clinical findings, histopathology features, DIF, and ELISA tests; this step-wise process is time-consuming.

Another major advantage of the biochip mosaic as a one step process is its cost. Currently, the biochip mosaic costs AU$37.76 (as of July 2017) to test one patient sample, but the new MesaCup Anti-skin Profile ELISA kit from MBL is AU$99.17 (as of July 2017). However, the ELISA kit includes an additional substrate, Type VII collagen, to diagnose epidermolysis bullosa, but the new biochip assay requires a separate slide. With regard to incubation times, the biochip requires the shortest incubation time of 1 hour in comparison with the MBL ELISA kit, which can take up to 2.5 hours; the EuroImmun ELISA kit takes 1.25 hours. Furthermore, the results from the biochip need an immunofluorescence microscope, but ELISA requires a spectrophotometric device to read the ELISA test results and thus can be used in small laboratories.

Nonetheless, contradictory studies have been published with regard to serum levels that reflect the clinical activity during the disease course. Some authors promote its use to monitor patient prognosis and treatment (Zhou et al., 2016), but others have observed no significant relationship between disease extent and levels of Dsg1 and Dsg3 autoantibodies (Bellon et al., 2014, Bracke et al., 2013). Although the biochip approach does not provide quantitative autoantibody serum levels like the ELISA methods, which have been shown to correlate with disease activity, the biochip can produce a semi-quantitative analysis by preparing various serial dilutions (1:10, 1:32, 1:100, 1:320, 1:1000, and 1:3200) and checking the highest dilution that still produces immunofluorescence (Russo et al., 2014, van Beek et al., 2012).

Furthermore, Barnadas et al. (2015) reported inaccuracies in anti-Dsg1 and Dsg3 ELISA values when titer levels exceeded 150 U/mL, which is possibly due to the saturation of the system and results in artefactually low scores. Additionally, later studies have demonstrated that a semi-quantitative analysis can be achieved. There have also been reports of high Dsg1 and Dsg3 ELISA values despite the absence of disease activity as well as low levels despite a relapse of pemphigus. However, these discrepancies may be explained by the fact that IgG autoantibodies that are directed against the N-terminus of Dsg1 and Dsg3 ectodomain EC1 appear to be more pathogenic than those that bind to epitopes on EC4 and EC5 (Amagai et al., 1992). Other targets, including acetylcholine receptors and pemphaxin, have also been shown to induce blisters in mice (Vu et al., 1998).

## Conclusion

The literature review demonstrates the comparable diagnostic accuracy of the biochip with the existing IIF and ELISA methods. The main advantages of the biochip that were identified include the simultaneous, multiparametric analysis of all relevant antibodies, which provides faster results and a more cost-effective and practical screening tool for patients with suspected autoimmune bullous dermatoses.

Common limitations that were identified in these studies include small sample sizes, low case-to-control ratios, and selection bias; however, there is still considerable evidence to support the use of biochip, mosaic-based, immunofluorescence testing. Future studies to address these issues are needed to further validate the use of the biochip in the routine diagnosis of pemphigus.

The biochip mosaic has the potential to be complemented with additional target antigens and accommodate up 16 biochips in a single incubation field. This could improve the accuracy and validity of the biochip; however, these substrates have yet to be included due to the rarity of the respective disorders.
